# Analysing public sector institutional capacity for health workforce governance in the South-East Asia region of WHO

**DOI:** 10.1186/s12960-019-0385-1

**Published:** 2019-06-18

**Authors:** Giorgio Cometto, Esther Nartey, Tomas Zapata, Mikiko Kanda, Yunus Md, Kavita Narayan, Kirana Pritasari, Aishath Irufa, Ramkrishna Lamichhane, Dileep De Silva, Thinakorn Noree

**Affiliations:** 1 Human Resources for Health Policies & Standards Health Workforce Department, World Health Organization, Avenue Appia 20, CH-1211 Geneva 27, Switzerland; 2 WHO, New Delhi, India; 3 Ministry of Health and Family Welfare, Dhaka, Bangladesh; 4 Ministry of Health and Family Welfare, New Delhi, India; 5 Ministry of Health, Jakarta, Indonesia; 6 Ministry of Health, Malé, Maldives; 7 Ministry of Health, Kathmandu, Nepal; 8 Ministry of Health, Nutrition and Indigenous Medicine, Colombo, Sri Lanka; 9 Ministry of Public Health, Bangkok, Thailand

**Keywords:** Health personnel, Health policy, Organization and administration

## Abstract

**Background:**

In order to analyse the institutional capacity for health workforce policy development and implementation in countries in the South-East Asia region, the WHO facilitated a cross-sectional analysis of functions performed, structure, personnel, management and information systems of human resources for health (HRH) units in Ministries of Health.

**Case presentation:**

A self-assessment survey on the characteristics and roles of HRH units was administered to relevant Government officials; the responses were validated through face-to-face workshops and by the WHO staff. Findings were tabulated to produce frequency distributions of the variables examined, and qualitative elements categorized according to a framework for capacity building in the health sector.

Ten countries out of the 11 in the region responded to the survey. Seven out of 10 reported having an HRH unit, though their scope, roles, capacity and size displayed considerable variability. Some functions (such as planning and health workforce data management) were reportedly carried out in all countries, while others (inter-sectoral coordination, research, labour relations) were only performed in few.

**Discussion and conclusions:**

The strengthening of the HRH governance capacity in countries should follow a logical hierarchy, identifying first and foremost the essential functions that the public sector is expected to perform to optimize HRH governance. The definition of expected roles and functions will in turn allow identifying the upstream system-wide factors and the downstream capacity requirements for the strengthening of the HRH units. The focus should ultimately be on ensuring that all the key strategic functions are performed to quality standards, irrespective of institutional arrangements.

**Electronic supplementary material:**

The online version of this article (10.1186/s12960-019-0385-1) contains supplementary material, which is available to authorized users.

## Background

For more than a decade, the World Health Organization (WHO) has recognized the need and advocated for greater focus on building or strengthening core institutional capacities for effective stewardship and governance of the health workforce agenda [1].

The WHO *Global Strategy on Human Resources for Health: Workforce 2030*, adopted by Member States at the World Health Assembly in May 2016, recognized the need “to build the capacity of institutions at sub-national, national and international levels for effective leadership and governance of actions on human resources for health.” [2]. Further, it called upon all countries to have a human resources for health (HRH) unit or department, noting that such a unit should have the capacity, responsibility, financing and accountability for a standard set of core functions of HRH policy, planning and governance, data management and reporting.

This need has been recognized also at the regional level: in the first WHO progress report on the Decade for health workforce strengthening in the South-East Asia region (SEAR) included a call “to document the existence and functions of HRH units in SEAR ministries of health” and “to provide technical assistance to Member States, including for strengthening governance and supporting HRH units to fulfil their functions” [3].

Also countries in the Asia and Pacific region prioritized improving HRH governance and creating and strengthen existing HRH units during the 9th Asia Pacific Action Alliance on Human Resources for Health (AAAH) conference held in Colombo in October 2016 [4].

The long-standing recognition of the importance of HRH governance and management contrasts with the reality and evidence of inadequate HRH planning [5], gaps in the translation of policy into implementation [6], and uneven application of efficient and ethical HRH management practices and behaviours [7]. Previous analyses have shed light on HRH governance by exploring several dimensions, including decision making, reforms and decentralization, partnership, equity and equality, among others [8]. However the discourse has not focused very explicitly on the role and functions of HRH units, which is the institutional locus with the responsibility in most Governments for tackling these challenges. In particular, the characteristics of HRH units are not widely documented, and evidence on factors that can optimize their performance is scanty. In order to analyse the institutional capacity and potential for strengthening of HRH units in the WHO Member States of the SEAR, the WHO conducted a cross-sectional analysis exploring the functions performed, structure, personnel, infrastructure and equipment, management and HRH information systems, the challenges faced and possible approaches to strengthen their role and performance. A better understanding of these factors, gained through the specific lens and focus on HRH units, can contribute to the wider discourse on the health workforce governance and management literature.

## Case presentation

### Methods

The assumption underpinning the conceptualization of this study is that a variety of factors may plausibly influence the effectiveness and performance of HRH units, including as follows: the institutional location of the HRH unit within the Government structure, the functions assigned, the number and qualification of personnel, availability of resources, continuity of staff, availability of formal planning documents and tools and mechanisms of interaction with owners of data.

A questionnaire (Additional file 1) was developed assessing these various domains through a mix of binary (yes/ no), multiple choice and open-ended questions, with the possibility of providing additional data and evidence as supplementary documents. The contents of the questionnaire were informed by the approach adopted in previous similar analyses [9], and its development followed an iterative consultation process involving the WHO staff responsible for HRH policy dialogue and technical support at the global and regional levels; the survey instrument was finally validated with the assistance of a senior Government official with extensive experience of leading and overseeing the work of an HRH unit in a different country than those in the region (see “Acknowledgements”).

Questions were organized into the following subgroups based on the scope of the inquiry: general information; functions; structure; personnel; infrastructure, equipment and operations; management, HRH information systems and coordination.

The survey was administered in the form of a self-assessment questionnaire to relevant focal points in governments of each of the eleven SEAR countries, who were identified through the WHO country offices. Preliminary and final findings were presented and discussed with Government focal points in workshops held in New Delhi, India, in September 2017 and April 2018, respectively. Subsequently, a more in-depth review and validation was conducted through dialogue between the WHO focal points at the country and regional levels and Government counterparts in the participating countries. The responses provided to the open-ended questions were later categorized according to commonly recurring themes for the purpose of analysis. Data was entered and tabulated using Excel software.

**Fig. 1:**
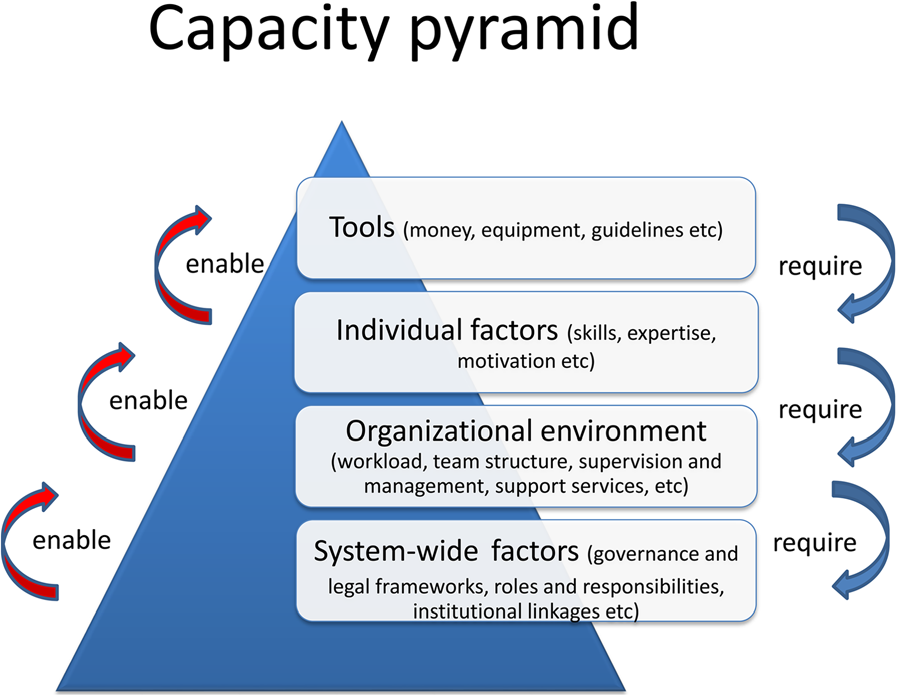
Health system workforce capacity pyramid (adapted from Potter and Brough)

The evidence gathered through the survey was categorized according to a framework, adapted from Potter and Brough [10], which enables characteristics of HRH units to be analysed according to system-wide, organizational and individual factors, in addition to the availability of specific resources and tools. This analytical lens recognizes a hierarchy of needs in capacity development, with elements at each level enabling and representing a pre-condition for the ones above (Fig. 1).

## Results

Ten countries out of eleven (Bangladesh, Bhutan, India, Indonesia, Maldives, Myanmar, Nepal, Sri Lanka, Thailand and Democratic Republic of Timor Leste) from the South-East Asia region of the WHO participated in the survey, providing their responses and subsequently validating them, in the period between September 2017 and September 2018.

The following sections provide both basic quantitative elements from the data collected and highlights of qualitative information emerging from the open-ended questions or the Additional files 1 and 2.

## System-wide factors

### Existence of an HRH unit and its location within the government structure

Of the 10 countries, 7 (70%) reported the presence of an HRH unit in their Ministry of Health, while the other 3 (30%) reported that different units/departments within their Ministry of Health were performing some HRH-related functions. Among the 7 countries with HRH units, 28.6% had been in existence in the preceding 5 years while more than half (57.1%) had been established for over 5 years.

The position of HRH unit heads within the governance frameworks and their professional backgrounds showed considerable heterogeneity (as shown in Table 1).

**Table 1:** Title and professional qualification of the heads of the HRH units

No	Title of HRH head	Professional qualification of head
1	Additional secretary	No response
2	Chief human resources officer	No response
3	1. Joint secretary 2. Technical advisor	1. Master of Technology 2. Master in Health Policy and Administration
4	No response	Dentist/Master of Science
5	Director—head of human resource division (temporary)	Bachelor’s Degree in Primary Health Care
6*	Director General	Expert in Public Health and Medical Education
7*	Joint secretary	Administration
8	Head	Board-certified consultant holding MD (either in Public Health or Medical Administration) Plus PhD in Health HR
9*	1. Director of human resource management division 2. Director of strategy and planning division	No response
10	Not indicated	Master of Public Health (MPH)

*Countries reporting not having an HRH unit

### Functions assigned to HRH units

All 10 countries reported the existence of a formal Government document on HRH responsibilities (whether or not these were performed by a single unit or distributed in different institutional loci), and 9 provided this document as evidence of its existence.

**Fig. 2:**
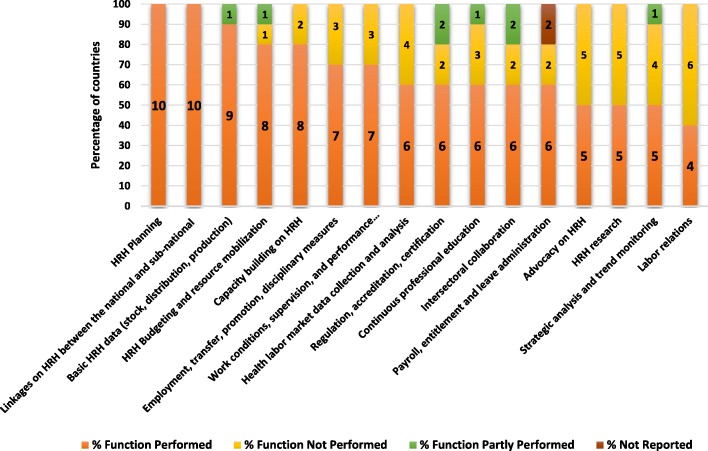
HRH functions performed in 10 countries included in this study

In terms of the assigned functions (Fig. 2), HRH planning and facilitation of appropriate linkages between the national and sub-national levels were reported by respondents in all 10 countries. Management of labour relations with health worker unions was performed in 4 (40%) countries.

Three of the 10 countries reported performing other functions, including training; review and approval of hospital capacity in case of upgrade request; curricula revision and update and deployment of recent scholarship graduates.

Various explanations were provided for not performing certain functions by HRH units in some countries, but most typically, the HRH function(s) not performed had either been assigned to other departments or were not being performed at all in the country. Examples of the latter included as follows: the coordination of an inter-sectoral national health workforce agenda; the facilitation of permanent mechanisms of collaboration among different stakeholders including the private sector; strategic analysis and monitoring of health workforce trends (including national and international mobility); and the contribution to management of labour relations with health workers’ unions/representatives.

## Findings on organizational environment

### HRH unit structure

All countries provided information on the job title of the official that the head of the HRH unit reported to, although it was not always clear from the organograms provided where the HRH unit was situated. Five (71.4%) out of the 7 countries reporting to have an HRH unit in MoH indicated having an organogram, with 4 (57.1%) providing it as attachments for evidence. Of the 3 countries not having an HRH unit in MoH, 2 (66.7%) had organograms with only one providing it as reference.

### Planning documents and management tools

**Fig. 3:**
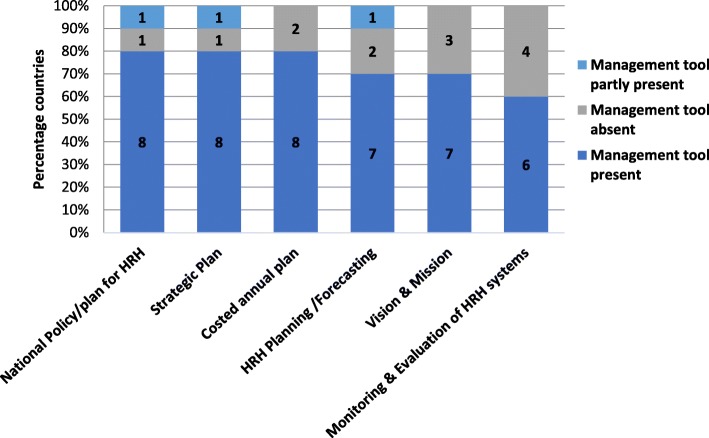
Availability of management tools

The most common management tools used by 80% of the responding countries were a strategic plan, national policy or plan for HRH, and a costed annual plan. However, monitoring and evaluation systems were absent in 40% of the countries (Fig. 3).

Eight out of the 10 countries included in this study indicated HRH areas prioritized in the preceding year (Table 2). HRH planning, data collation, regulation, education and capacity building for health workers were activities prioritized across countries; conversely, no country reported prioritizing, in the year preceding the survey, activities on inter-sectoral collaboration, linkages between the national and sub-national administration, strategic analysis and trend monitoring, HRH research, payroll, entitlement and leave administration, decisions on employment, transfer, promotion, disciplinary measures for staff, advocacy of HRH and labour relations.

**Table 2:** Work areas prioritized by HRH unit during year preceding the survey compared to functions assigned to HRH unit

Functions assigned	Work areas prioritized
HRH planning	Work load assessment; planning/forecasting projections for HRH demand/supply for the country; development of HRH strategic roadmap 2030 or strategic plan; health workforce plan
HRH budgeting and resource mobilization	Annual budget
Work conditions, supervision and performance appraisal mechanisms	Performance appraisal system; inspection trips and audit visits
Basic HRH data (stock, distribution)	Development of HRH registry; implement of human resources database
Health labour market data	Fulfillment of health human resources needs in remote areas, borders and outer islands; recruitment and retention; nursing and midwifery field
Regulation, accreditation, certification	Focus on unregulated professionals (excluding frontline health workers)
Continuous professional education	Training and development/training requirements/in-service training scholarship
Capacity building on HRH	Capacity building of staff

**Fig. 4:**
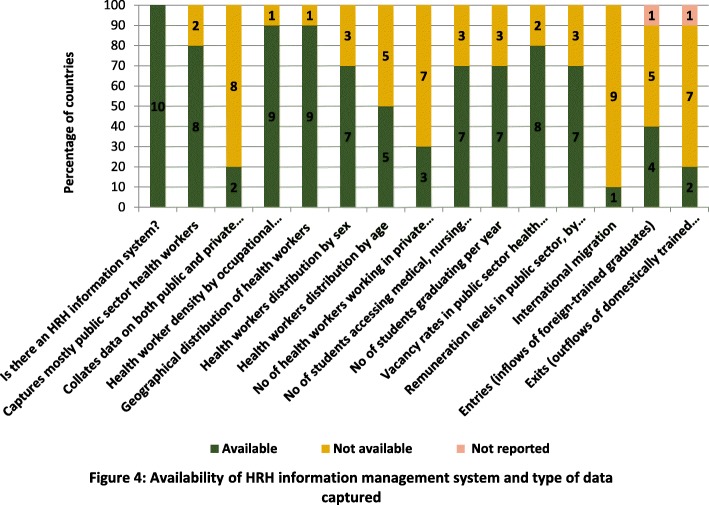
Availability of HRH information management system and type of data captured

All 10 countries reported that they had an HRH information system. The HRH information system captures mostly information on public sector health workers in 80% of the countries and collates data from both private and public health sector workers in 20% of the countries (Fig. 4). Other data captured in the HRH information system included health worker density by occupational category and geographical distribution of health workers (in 90% of the countries). Data least available in the HRH information system was information on the number of health workers in the private sector (captured in 30% of the countries) and data on international migration (captured in 10% of the countries) (Fig. 4).

### Mechanism of interaction with owners of data

**Fig. 5:**
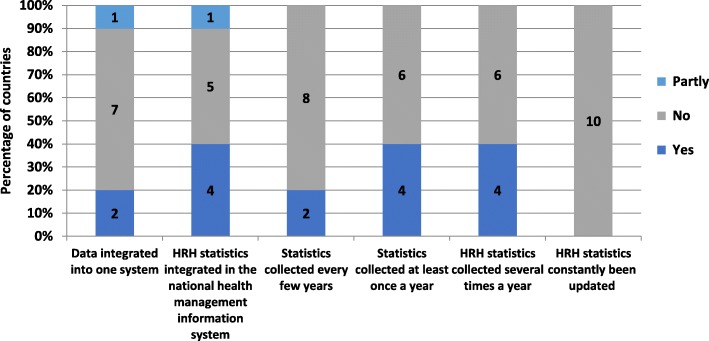
HRH data collection and integration

Seven out of the 10 countries reported that HRH data were not integrated into one system. In one of the countries, this process was reported to be on-going. However, none, out of the 10 countries, had their HRH statistics been constantly updated through a live workforce registry. Collection of data was reported to be conducted several times in the year by 4 (40%) countries and once a year by another 4 (40%) countries (Fig. 5).

Five (50%) of the countries participating in the survey reported that HRH units performed a validation of HRH data. Data validation methodologies included stakeholder consultations, performing of quality checks on the data, automatic validation though the management information system, HRH coding, use of excel and performing manual checks on the data. The methods of analysis of HRH data reported by respondents include, among others, disaggregation of data by sex, geographical distribution and occupational group. Out of the 10 countries, HRH statistics were publicly available in 7 (70%) and included in a yearly MoH publication in 8 (80%) of the countries.

## Findings on individual factors

### The number and qualification of personnel

The total number of staff working in the units performing HRH functions was reported to be 15 and above in 40% of the countries and less than 5 in 20% of the countries (Table 3A). Out of a total of 594 staff reported to be working in the HRH units across these 10 countries, administrative staff constituted 59%, whereas professional staff formed 14% and other staff (e.g. ancillary and support workers) 27% (Table 3B).

**Table 3:** Characteristics of HRH units

A: Total no. of staff working in HRH unit		B: Skills composition of staff		C: No. of heads in the last 5 years		D: Tenure of HRH heads	
No. of staff	No. of countries *n* (%)	Category of staff	No. of staff *n* (%)	No. of heads	No. of countries *n* (%)	Months	No. of countries *n* (%)
< 5	2 (20)	Professional staff	86 (14)	0–2	4 (40)	< 24	7 (70)
5–9	0 (0)	Administrative staff	347 (59)	3–5	3 (30)	24–48	2 (20)
10–14	2 (20)	Other staff	159 (27)	> 5	1 (10)	Not reported	1 (10)
15 and above	4 (40)	Total no. of staff	594 (100)	Not reported	2 (20)		
Not reported	2 (20)						

**Fig. 6:**
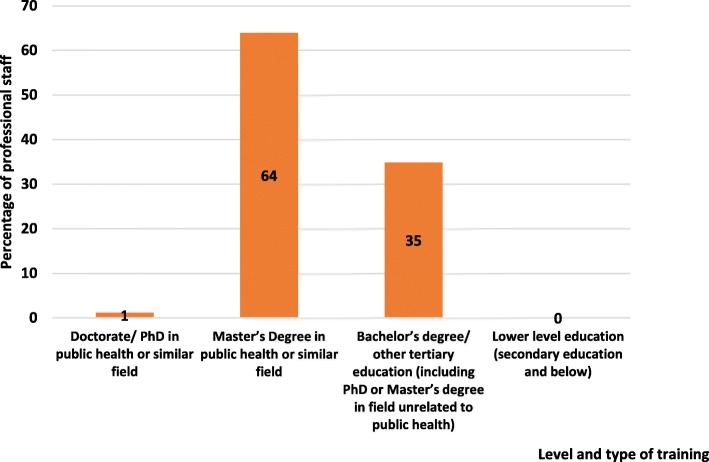
Level and type of training of professional staff in HRH units

Most professional staff of HRH units held a Master’s Degree in Public Health or a similar field (Fig. 6).

### Continuity of service for heads of HRH units

Four [4] countries reported their HRH units to have had 2 heads within the last 5 years. One country however did have more than five [5] heads within the preceding 5 years (Table 3C).

In 7 (70%) of the countries, HRH heads had been in their role for less than 24 months (Table 3D.). The current head of the unit was reported to have worked on HRH issues for less than 24 months in 4 (40%) of the countries, and more than 48 months in 2 (20%).

## Findings on tools

### Availability of resources

In 5 of the 10 countries, office space available to the HRH units was less than 60 m^2^, while in 2 countries, it was more than 120 m^2^. Data were stored both on paper and electronically in 70% of the countries while one country used only paper as the means of storage of data (10%).

Six of the countries reported that their HRH units had more than 10 functioning computers. However, with regard to functioning laptops, only 1 country had more than 10 functioning laptops. All the HRH units within the 10 countries had internet connection.

### Financial resources

**Fig. 7:**
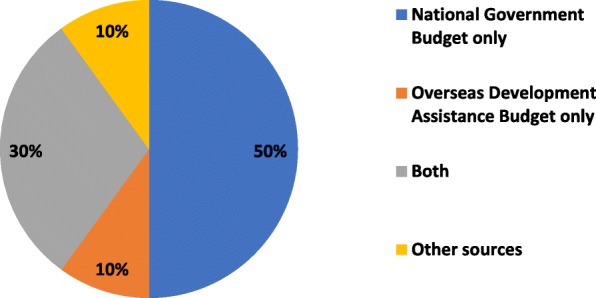
Sources of financing for HRH units

Funding for HRH units was reported to originate from domestic, overseas development or mixed sources (Fig. 7).

**Fig. 8:**
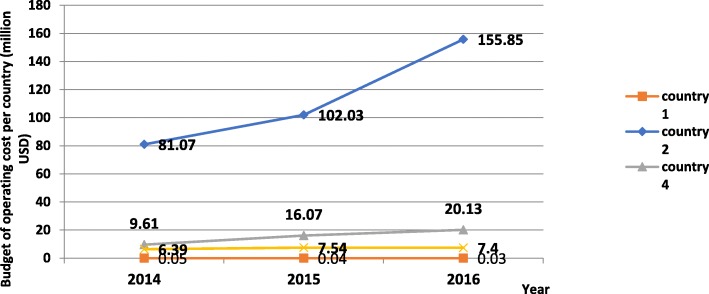
Budget of operating cost for HRH units in the preceding 3 years

Four countries out of 10 reported the operating budget (Fig. 8) and 3 out of 10 the expenses of the HRH unit in the 3 years preceding the survey (2014–2016). In 2016, budget for HRH units ranged from 0.03 to 156 million dollars. The high variability relates primarily to the variance in the country population and overall health budget, in addition to the functions assigned to the HRH units.

## Cross-cutting issues

Various policy and operational challenges affecting the performance of HRH units were mentioned by the respondents in the open-ended questions, including:

- System-wide factors: need to reinforce leadership and political commitment;
- Institutional: HRH functions scattered among different divisions in MoH, with limited institutional linkages and coordination mechanisms for policy coherence and data sharing;
- Organizational environment: inadequate supervision and management, weak regulatory environment and lack of disciplinary measures, unclear functional responsibilities;
- Individual factors: lack of motivation, lack of expertise in human resources development and public health;
- Tools: poor availability of financial resources.

Both generic and specific solutions were proposed for some of the most prevalent challenges. Examples of proposed solutions specific to HRH governance and leadership included strengthening the professional qualifications and skills of HRH unit staff at the individual level; establishing a central HRH department where HRH functions could be combined and/ or coordinated; strengthening coordination mechanisms and cooperation with relevant stakeholders in health and other sectors and creating mechanisms for regular sharing of HRH data.

## Discussion and conclusions

### Limitations

This study, conducted through a self-assessment survey, was prone to subjectivity, possible variations in interpretation of the questions and potential respondent bias. To mitigate these risks, two strategies were adopted: (1) the preliminary findings were discussed during a face-to-face workshop, which provided an opportunity to clarify scope of the study and the precise nature and expectation of the questions in the survey and (2) the technical contents of the responses were validated with the support of the WHO staff.

The survey can be considered representative of challenges and opportunities faced by HRH units (or equivalent mechanisms and bodies) in the SEAR. Caution should however be exercised in extrapolating findings to other regions. Data provided through the questionnaires were generally complete, but data on budget and expenditures by HRH units were more limited.

The relatively little sample size (10 countries) limited the scope for analyses exploring the existence of correlations between specific characteristics of HRH units and HRH-related outcomes that could be plausibly associated a priori with effective HRH governance.

### Interpretation of findings

Despite these limitations, the analysis was able to assess the HRH units at the national level of countries in the region.

Most countries (70%) indicated having had an HRH unit for more than 5 years. Having a single HRH unit, however, is not the only possible governance arrangement: some respondents reported a fragmentation of the HRH agenda linked to the scattering of responsibilities across different functional units and departments. In the aggregate, these findings would support the interpretation that a central HRH unit would probably be advantageous in most contexts, but that its role can vary and include both models in which it performs directly most or all of the relevant HRH governance functions or—if these are by statute allocated to other units—at the very least performs a coordination role.

In most countries, it was reported that the HRH units reported to a senior level (Director General or Permanent Secretary) in the overall MoH organogram, indicating that, at least in theory, they should have an opportunity to have direct access to the decision-making level. Some responses provided through the questionnaires however indicated that strengthened communication and advocacy would be required to secure the required political support.

In terms of functions performed, the survey indicated that all or the vast majority of countries performed at least some of the core functions, such as HRH planning, policy development and basic HRH data management; other functions were carried out by other departments and mechanisms. More concerning was the finding that some functions were not performed at all in several countries, including, e.g. inter-sectoral and multi-constituency coordination research and monitoring of trends, labour relations with health workers’ representatives. These roles are critical to improve policy dialogue in countries, especially with regard to a more strategic and long-term orientation of the HRH agenda, built and implemented in partnership with other relevant sectors, including education, finance and labour. These findings echo those of earlier literature, which identified examples of inter-sectoral coordination mechanisms to have a positive effect on health workforce governance [11, 12], as well as the importance of building effective partnerships with development partners [13, 14] and the private sector [15, 16].

Therefore, while some flexibility in the structures of HRH units is appropriate, deliberate efforts should be made to ensure that all core functions be performed, irrespective of whether this happens in a single unit or department or as a result of effective coordination across different ones.

Performing effectively the roles assigned requires that HRH units have adequate capacity and resources: the findings of the survey reveal that some HRH units had a very limited staffing complement, while others a relatively large one. This heterogeneity in size most probably reflects differences in the population size of countries and in roles, both in respect of functions and in terms of different division of responsibilities between units at the national level vis-a-vis equivalent bodies at the sub-national levels in the context of decentralized or devolved health systems, which is the typical situation in large and/ or federal countries. The opportunities, implications and drawbacks of decentralization of HRH functions have been discussed elsewhere [17–19]. But there is a need, across different country contexts, to align staffing of HRH units with expected functions; an appropriate functional allocation of staff is as important as the overall size and composition.

In some of the countries, a relatively short average tenure of the head of the HRH unit may contribute to rapidly shifting priorities, institutional instability and inconsistent pursuit of policy objectives. These findings reinforce a concern that HRH planning and development may still largely be seen as a routine administrative function, subject to a rapid turn-over, often as a part of routine civil service rotation schemes. Many respondents reported gaps in the HRH units in terms of specific technical capacity on HRH; conversely, HRH should be best understood as a specialized technical area of public health, which requires years of experience and ideally dedicated training [20]. Experiences from other contexts underscore the importance of adequate management competencies to ensure the successful design and implementation of HRH policies to improve both health outcomes and harness the employment creation potential of the health sector [21, 22]. The findings reinforce a need to both strengthen the technical profile of HRH units by increasing the relative proportion of professionals employed and to create an empowering and rewarding work environment that, like for the rest of the health workforce, can foster the attraction and retention of talent [23].

While a basic HRH information system was reported to be functioning by most countries, data were most typically limited to public sector health workers, and there were substantial limitations found in the systems for HRH data validation, integration and analysis. The mandate, capacity and information systems of HRH units and of national HRH information systems should therefore be expanded to more explicitly include maintaining an overview of strategic information and intelligence on the health workforce at large, whether employed by the public or private sector [24]. The routine implementation of national health workforce accounts provides a comprehensive framework to advance this agenda [25].

The availability of material inputs (office space, computers, etc.) did not appear to be a major area of concern in the countries of this region, but availability of adequate financial resources was often reported as insufficient. Given the multiplier effect that the work of HRH units can have through the design and implementation of more cost-effective health workforce policies, their work should be adequately funded and include flexibility to fund priority activities, in addition to fixed recurrent costs (such as salaries).

The reported high dependence on external funding in some countries represented an additional concern, with a risk of displacing domestic investments, volatility of support and sustainability [26]. But in the case of external dependence of core stewardship functions expected of a Ministry of Health—as HRH governance and policy setting undoubtedly is—there is an additional layer of risk in terms of a distortion by external actors of national priority-setting processes and reduced government accountability to its own citizens [27]. National Governments should invest adequate domestic resources in their HRH units and put in place safeguards to guarantee their technical autonomy and financial and programmatic independence from external partners.

Workshops were organized in September 2017 and April 2018 to review the preliminary findings of the study and discuss the policy implications. Participating countries welcomed the findings of the study and are considering a number of policy options to improve HRH governance in their countries: two of the three countries that reported not having an HRH unit recognized the importance of having one and are currently in the process of setting it up. Most of the SEAR countries that reported having an HRH unit identified a need to strengthen the capacity of the staff—in both quantitative and qualitative terms—and to overcome the high turn-over affecting leadership positions in the HRH units. At the governance level, countries recognized the importance of creating inter-sectoral coordination mechanisms to be more effective in HRH planning.

### Policy implications

The findings of this study can contribute to the broader conceptualization of capacity building initiatives on the HRH agenda in SEAR.

The strengthening of the HRH governance capacity should follow a logical hierarchy, identifying first and foremost the essential functions that the public sector is expected to perform to optimize HRH governance. The definition of expected roles and functions will in turn allow to identify the upstream system-wide factors and the downstream capacity requirements for the strengthening of the HRH units. Among the former, a fundamental enabler is to build political ownership and support for reform and capacity building on HRH governance [28].

The broader spectrum of factors that determine the outcomes of health policy making in the HRH domain should be considered: health policy making the health workforce domain is influenced by factors linked to health needs, fiscal space, economic policy, employment and labour policies and risks to trigger industrial actions by health workers. However, since opportunities for policy change stem from the iterative relations among the three processes of identifying problems, developing relevant technical solutions and building policy support for the latter [29], the importance of building sound capacity for analysis of HRH challenges and proposal of appropriate solutions should not be underestimated.

The role and function of the HRH unit should be considered within the unique institutional and governance framework of each country.

Effectively performing the existing functions and considering an expansion of the scope of work of HRH units will however necessitate a staffing complement commensurate to the roles, in terms of both numbers and skills, selected based on skills and merits, and receiving adequate support.

To date, despite a wide recognition of the importance of the health workforce, relatively few experiences have been documented on improving HRH governance, leaving many policy questions unanswered. The findings from this survey shed some light on existing capacity and gaps in public sector HRH governance in the SEAR, but also underscore the importance of further documenting experiences, understanding the political economy of HRH policy making and creating opportunities for mutual learning [30].

## Additional files

Additional file 1: Self-assessment questionnaire for HRH units. (DOCX 22 kb)

Additional file 2: HRH survey SEARO data collection. (XLSX 659 kb)

## Data Availability

The database with all the available information and data has been annexed to the submission.

## Abbreviations

HRH: Human resources for health
MoH: Ministry of Health
MPH: Master in Public Health
PhD: Philosophy Doctor
SEAR: South-East Asia region
WHO: World Health Organization
