# Supplementary material for: Analysing public sector institutional capacity for health workforce governance in the South-East Asia region of WHO
Source: Hum Resour Health. 2019 Jun 18;17:43. doi: 10.1186/s12960-019-0385-1 (PMC6582590; doi:10.1186/s12960-019-0385-1)
Supplement: Supplementary file 1 — Self-assessment questionnaire for HRH units. (DOCX 22 kb) [file 12960_2019_385_MOESM1_ESM.docx]

# Additional file 1: Self-assessment questionnaire for HRH units*.*

| **A. General information** | | | | | | | | | | | | | |
| --- | --- | --- | --- | --- | --- | --- | --- | --- | --- | --- | --- | --- | --- |
| 1. Country |  | | | | | | | | | | | | |
| 1. Date |  | | | | | | | | | | | | |
| 1. Name and contact information (e-mail/ phone) of the questionnaire respondent |  | | | | | | | | | | | | |
| 1. Position of the questionnaire respondent |  | | | | | | | | | | | | |
| 1. Is there a HRH unit in the Ministry of Health or a similar body (in other Ministry or autonomous? | □ Yes, in MoH  □ Yes, in a different Government structure (specify _______________)  □ No, but different HRH-related functions are performed by different units/ departments  (provide details _______________________________________)  --🡪 complete the rest of the questionnaire with responses that reflect the combined functions, staff and structures of the relevant units  □ No --🡪 go to question 30 | | | | | | | | | | | | |
| 1. Name of the HRH unit |  | | | | | | | | | | | | |
| 1. When was the HRH unit established? | _______ (month/year) | | | | | | | | | | | | |
| 1. Title and professional qualification of the head of the HRH unit. |  | | | | | | | | | | | | |
| **B. Functions** | | | | | | | | | | | | | |
| 1. Is there a formal/ written document defining the tasks/ responsibilities of the HRH unit and its structure? | □ Yes (please attach or provide the web link to the document)  □ No | | | | | | | | | | | | |
| 1. When was the document prepared/ last updated? | Prepared (month/year) _________  Last update (month/year) ________ | | | | | | | | | | | | |
| 1. Which of the following functions are carried out by the HRH unit? | Functions: | | | | | | | | | | Yes / No | | |
|  | **HRH policy and strategy** | | | | | | | | | |  | | |
|  | 11.1 Development of policies, strategies, plans, frameworks for HRH; | | | | | | | | | | □ □ | | |
|  | 11.2 Coordinate an inter-sectoral national health workforce agenda, facilitating permanent mechanisms of collaboration among different line ministries (education, finance, labour…) including the private sector; | | | | | | | | | | □ □ | | |
|  | 11.3 Facilitate appropriate linkages on HRH between the national and sub-national administration; | | | | | | | | | | □ □ | | |
|  | 11.4 Budgetary and resource planning and mobilization for HRH, in collaboration with ministries of finance and other relevant constituencies; | | | | | | | | | | □ □ | | |
|  | 11.5 Define job descriptions, working conditions, supervisory and performance appraisal mechanisms, reward systems and career structures for health workers, in collaboration with relevant civil service bodies; | | | | | | | | | | □ □ | | |
|  | **HRH data, information and evidence** | | | | | | | | | | | | |
|  | 11.6 Collection and analysis of health workforce data (e.g. stock, distribution, production, etc); | | | | | | | | | | □ □  □ □ | | |
|  | 11.7 Collection and analysis of health labour market data (e.g. employment status, vacancies, etc.) | | | | | | | | | | □ □ | | |
|  | 11.8 Strategic analysis and monitoring of health workforce trends (including national and international mobility); | | | | | | | | | | □ □ | | |
|  | 11.9 Stewardship of a national agenda on HRH research, including publication and dissemination of good practices; | | | | | | | | | | □ □ | | |
|  | **HRH accreditation, education and training** | | | | | | | | | | | | |
|  | 11.10 Set policies on regulation, accreditation, certification, education, in collaboration with professional councils and academic institutions; | | | | | | | | | | □ □ | | |
|  | 11.11 Continuing professional development for health workers; | | | | | | | | | | □ □ | | |
|  | **HRH leadership, advocacy and policy dialogue** | | | | | | | | | | | | |
|  | 11.12 Build capacity for HRH policy and management | | | | | | | | | | □ □ | | |
|  | 11.13 Advocate for HRH investments and health workers’ rights and working conditions | | | | | | | | | | □ □ | | |
|  | 11.14 Contribute to management of labour relations with health workers’ unions/ representatives | | | | | | | | | | □ □ | | |
|  | **HR administration and management for health workers** | | | | | | | | | | | | |
|  | 11.15 Payroll, entitlements and leave administration; | | | | | | | | | | | | |
|  | 11.16 Decisions on employment, transfer, promotion, disciplinary measures for staff. | | | | | | | | | | □ □ | | |
| 1. Does the HRH unit perform other functions? If yes please explain. |  | | | | | | | | | | | | |
| 1. If you answered “NO” to any of the functions in question 11 above, please explain if these functions are carried out by another unit/ department/ entity, if they are performed at a different level (e.g. at sub-national level), or if they are not performed in your country. | 11.1  11.2  11.3  ….  11.16 | | | | | | | | | | | | |
| **C. Structure** | | | | | | | | | | | | | |
| 1. To whom does the HRH unit head report to? (indicate level in the organogram and professional title and name of division/ directorate/ office of the Permanent Secretary/ Director General or equivalent). |  | | | | | | | | | | | | |
| 1. Does the HRH unit have an organogram? If so please attach. | □ No  □ Yes (please attach or provide the web link to the organogram) | | | | | | | | | | | | |
| **D. Personnel** | | | | | | | | | | | | | |
| 1. How many staff work in the unit? Provide details on skills composition of HRH unit. Disaggregated statistics by level and type of training. | Doctorate/ PhD in public health or similar field | | Master’s Degree in public health or similar field | | Bachelor’s degree/ other tertiary education (including PhD or Master’s degree in field unrelated to public health) | | Lower level education (secondary education and below) | | | | | | Total |
| Professional |  | |  | |  | |  | | | | | |  |
| Administrative |  | |  | |  | |  | | | | | |  |
| Other (support staff) |  | |  | |  | |  | | | | | |  |
| Grand total |  | |  | |  | |  | | | | | |  |
| 1. How many heads/ directors has the HRH unit had in the last 5 years? | ______ | | | | | | | | | | | | |
| 1. How many years and months has the current head of the unit been in her/ his post? | ___ /____ (months/years) | | | | | | | | | | | | |
| 1. How many years and months has the current head of the unit worked on HRH issues? | ___ /____ (months/years) | | | | | | | | | | | | |
| **E. Infrastructure, equipment and operations** | | | | | | | | | | | | | |
| 1. What is the office space (in square meters) available to the HRH unit? (approximate values) |  | | | | | | | | | | | | |
| 1. How does the unit keep its data? | □ On paper  □ Electronically | | | | | | | | | | | | |
| 1. How many functioning computers/laptops does the HRH unit have? |  | | | | | | | | | | | | |
| 1. Does the unit have an internet connection? | □ No  □ Yes | | | | | | | | | | | | |
| 1. Do all professional and administrative unit staff have an internet connection? | □ No  □ Yes | | | | | | | | | | | | |
| 1. Do all professional and administrative unit staff have access to a telephone connection? | □ No  □ Yes | | | | | | | | | | | | |
| 1. What was the budget for operating costs of the unit in the preceding 3 years? Please use US $ | 2014 | | | 2015 | | | | 2016 | | | | | |
|  | Budget | Expenditure | | Budget | | Expenditure | | | Budget | | | Expenditure | |
|  |  |  | |  | |  | | |  | | |  | |
| 1. What are the main sources of financing for the operations of the HRH unit? | □ National Government budget  □ Overseas Development Assistance budget  □ Other: ________________________ | | | | | | | | | | | | |
| **F. Management, HRH information system and coordination** | | | | | | | | | | | | | |
| 1. Does the unit have/ use/ implement any of the following management tools? If so please attach or provide the web link. |  | | | | | | | | | Yes/ no | | | |
|  | Vision and mission | | | | | | | | | □ □ | | | |
|  | Strategic plan | | | | | | | | | □ □ | | | |
|  | Costed annual plan of operations | | | | | | | | | □ □ | | | |
|  | Planning/ forecasting projections for HRH demand/ supply for the country | | | | | | | | | □ □ | | | |
|  | Monitoring and evaluation systems | | | | | | | | | □ □ | | | |
|  | Other | | | | | | | | | □ □ | | | |
| 1. Which work areas were prioritized by the HRH unit last year? |  | | | | | | | | | | | | |
| 1. What data are available in the national HRH information system? | □ Health workers density by occupational category  □ Geographical distribution of health workers  □ Health workers distribution by sex  □ Health workers distribution by age  □ Number of health workers working in private sector  □ Number of students accessing medical, nursing schools per year  □ Number of students graduating per year (doctors, nurses and midwives, dentists, pharmacists, others)  □ Vacancy rates in public sector health facilities  □ Remuneration levels in public sector, by cadre  □ International migration data  □ Entries (inflows of foreign-trained graduates)  □ Exits (outflows of domestically trained graduates) | | | | | | | | | | | | |
| 1. Is all the HRH data integrated into one system? | □ No  □ Yes | | | | | | | | | | | | |
| 1. How often are HRH statistics collected? | □ Every few years  □ At least once a year  □ Several times a year (e.g. quarterly)  □ HRH data are constantly updated through a live workforce registry | | | | | | | | | | | | |
| 1. Is there a process to validate HRH statistics? Please describe |  | | | | | | | | | | | | |
| 1. How are HRH statistics analysed? |  | | | | | | | | | | | | |
| 1. Are the HRH statistics integrated in the national health management information system? | □ No  □ Yes | | | | | | | | | | | | |
| 1. Are HRH statistics publicly available? included in a yearly MoH publication/ progress report? | Publicly available  □ No  □ Yes  Included in yearly MoH publication  □ No  □ Yes | | | | | | | | | | | | |
| 1. Do you have any comments on the HRH unit and the environment in which it operates that were not captured in the preceding sections of this questionnaire? |  | | | | | | | | | | | | |
| 1. Is there a national policy/ plan for HRH? | □ No  □ Yes (please attach or provide the web link) | | | | | | | | |  | | | |
| 1. Does the HRH information system include both public and private sector health workforce? | □ No HRH information system exists  □ The HRH information system is largely dependent on the public sector payroll, therefore it captures mostly public sector health workers  □ Yes, the HRH information system has mechanisms to collate data on both public and private sector health workers _____________________________________________________________________________________________________________) | | | | | | | | | | | | |
| 1. Are there particular challenges or problems in HRH policy and management in your country? |  | | | | | | | | | | | | |
| 1. Do you have any comments on how HRH management in your country could be further improved and streamlined? |  | | | | | | | | | | | | |
